# The Role of Oxygen Tension in Penile Erection and Its Relationship to Erectile Dysfunction

**DOI:** 10.1100/tsw.2004.77

**Published:** 2004-09-13

**Authors:** Jong-Kwan Park, Robert B. Moreland, Ajay Nehra

**Affiliations:** ^1^ Department of Urology; Mayo Clinic and Foundation, and Mayo Medical School, Rochester, Minnesota 55905, USA; ^2^ Departments of Urology and Physiology, Boston University School of Medicine, Boston, Massachusetts 02118, USA

## Abstract

The corpus cavernosum of the penis is one of the few vascular beds in which there is a change in oxygen tension with function (blood PO_2_ 25-40mm Hg in the flaccid state, and 90-100mm Hg in the erect state). This change in oxygen tension exposes the components of the corpus cavernosum to a variety of cytokines, humoral, vasoactive, and growth factors which may affect the structure and function of the endothelial cells, smooth muscle cells, neurons and extracellular matrix. Among these cell types, endothelial cells are the first line of defense to blood-borne stress and can affect the underlying smooth muscle via paracrine mechanisms. Impotence is defined as the inability to obtain or sustain an erection sufficient for vaginal penetration and can result from a variety of pathological conditions, vascular disease, endocrine disease, neurological disease, and psychogenic disorders. The penis is a vascular organ and as such is susceptible to the effects of vascular diseases. This review will discuss the basic etiology of erection and vasculogenic erectile dysfunction and explore the role oxygen tension in regulating various cellular and humoral factors as well as trabecular structure and function.

## PENILE ANATOMY AND THE PHYSIOLOGY OF ERECTION

The penis is composed of three bodies of erectile tissue; a pair of corpora cavernosa and the corpus spongiosum which surrounds the urethra and terminates in the glans penis[1]. The corpora cavernosa are encompassed by a thick collagenous band, the tunica albugenia. During erection, the penis acts as a fluid-filled capacitor, accumulating blood under pressure[2,3]. Dilation of the resistance arterial bed of the penis allows transmission of systemic blood pressure to the corpora. Subsequent relaxation of the trabecular smooth muscle results in the expansion of the lacunar spaces and subsequent trapping of blood because of the elongation and compression of the draining venues. The helicine arterioles, multiple cork-screw shaped blood vessels, branch off the deep penile cavernosal artery and open directly into the lacunar spaces where they act as resistance arteries controlling the delivery of flow and pressure to the individual corpora of the corpora cavernosa1. Vasoconstriction of these arteries generates a large pressure gradient between the deep penile cavernosal artery and the lacunar spaces, which may be necessary to maintain the flaccid state of the penis[2].

At flaccidity, the trabecular smooth muscle of the corpus cavernosa as well as the helicine resistance arterioles are contracted; principally by alpha 1 and alpha 2 adrenergic mechanisms (Figure 1)[1]. Upon stimulation, nitric oxide (NO) is released from non-adrenergic, noncholinergic (NANC) nerve terminals and diffuses into the underlying smooth muscle in the helicine resistance arterioles as well as the trabecular smooth muscle of the corpus cavernosum[1,4]. NO binds to the heme of soluble guanylate cyclase in the smooth muscle cells, stimulating the synthesis of cGMP. As a result of increased cGMP synthesis, intracellular calcium levels decrease, and smooth muscle relaxation ensues. Other vasodilators such as vasoactive intestinal peptide (VIP) and calcitonin gene-related peptide (CGRP) may also be released from nerve terminals and trigger smooth muscle relaxation via specific receptors and increasing intracellular cAMP synthesis[1].

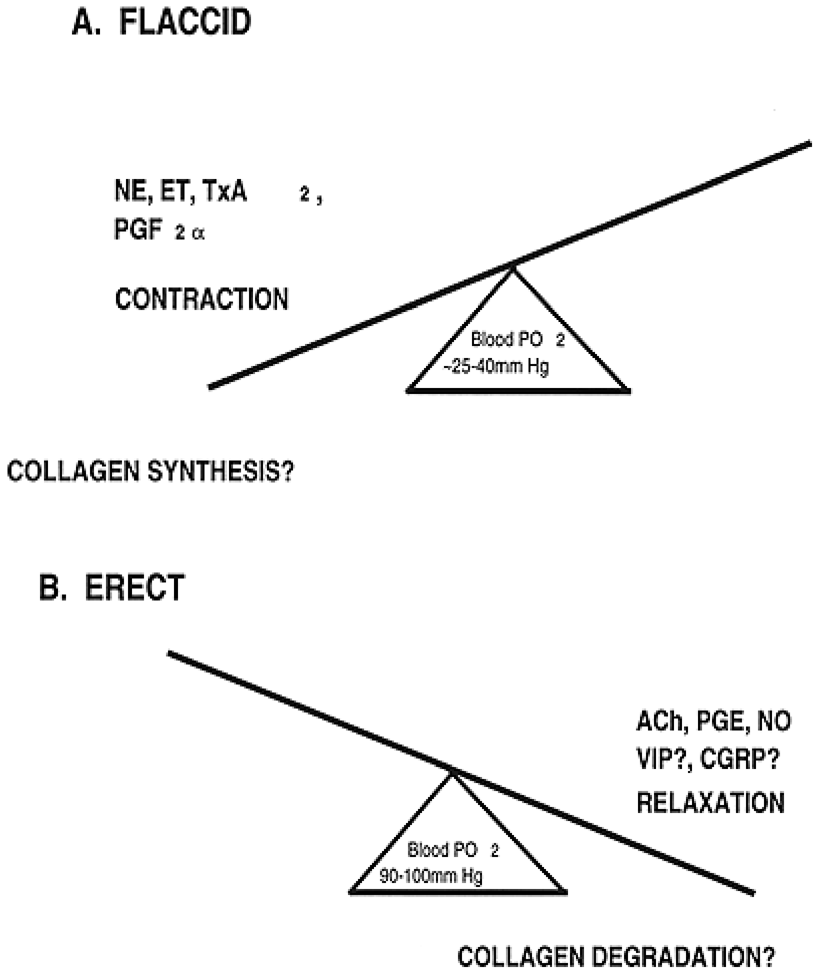

**FIGURE 1**. The seesaw of erectile (dys)function. Panel A: At flaccidity, corpus cavernosal blood PO2 is low (25-40mm Hg), the trabecular smooth muscle of the lacunar spaces contracted. It is our hypothesis that these conditons favor the production of growth factors, cytokines, and vasoactive factors which favor connective tissue synthesis. Panel B: During erection, corpus cavernosal blood PO2 increases and plateaus at 90-100mm Hg and the trabecular smooth muscle of the lacunar spaces relaxes. We further hypothesize that these conditions favor connective tissue degradation. It is the sum of both processes which lead to the trabecular smooth muscle/connective tissue balance (Adapted from Reference 3).

As the helicine arterioles dilate, an influx of oxygenated, arterial blood enters the corpora cavernosa; activating the endothelial nitric oxide synthase[1,4]. This endothelial-derived NO diffuses into the smooth muscle, further enhancing relaxation. Prostaglandin E is synthesized by the smooth muscle cells[5] and may further enhance smooth muscle relaxation in an autocrine fashion by binding to specific PGE (EP2 and/or EP4) receptors on the surface of the smooth muscle cells and elevating cAMP synthesis[5-7]. Smooth muscle interconnections via gap junctions may also enhance rapid propagation of the signal as they allow passage of molecules such as cyclic nucleotides and ions between cells, forming a functional synctium[1,8]. As the corpora expand outward and fill with blood, the draining venules are pressed against the tunica albugenia and occluded. The blockage of outflow results in veno-occlusion; corporal pressure rises to ~100mm Hg and erection ensues. Detumescence, while not as well characterized, is thought to involve reestablishment of the alpha adrenergic contraction of the corpora cavernosa[1,8].

## OXYGEN TENSION MEDIATES PENILE ERECTION

Despite the arterial blood supply via the helicine resistance arterioles, at flaccidity, the penile blood $PO_2$ is 25-40mm Hg and the corpus cavernosum trabecular smooth muscle is contracted[9-11]. During erection, the trabecular smooth muscle relaxes, allowing an increased inflow of arterial blood, and the cavernosal blood $PO_2$ increases and reaching a plateau at 90-100mm Hg[9-11]. This increase in blood $PO_2$ precedes the rise in cavernosal pressure which represents functional veno-occlusion as the subtunical draining venules are stretched and occluded. Recent studies suggests these changes in oxygen tension play an active role in regulating penile erection. At low oxygen tensions, measured in the flaccid state of the penis, the synthesis of nitric oxide is inhibited, preventing trabecular smooth muscle relaxation[9]. This inhibition of nitric oxide synthesis is probably necessary for the maintenance of penile flaccidity[4,9]. Following vasodilatation of the resistance arteries, the increase in arterial flow raises oxygen tension. In the oxygen enhanced environment, autonomic dilator nerves and the endothelium are able to synthesize nitric oxide, mediating trabecular smooth muscle relaxation. Therefore, physiologically, oxygen tension may regulate the types of vasoactive substances present in this vascular bed; *i.e.* at low oxygen tensions, vasoconstrictors (such as norepinephrine and endothelin) may predominate, while at high oxygen tension, nitric oxide as well as PGE are made *in sit*u. It is our hypothesis the difference in oxygen tension in the flaccid and erect states results in the regulation of synthesis of cytokines, autacoids, growth and vasoactive factors, which play a major role not only in trabecular smooth muscle tone but in connective tissue metabolism as well (Figure 1)[12]. Thus, in flaccidity, reduced oxygen tension leads to trabecular connective tissue synthesis and during erection, increased oxygen tension leads to reduced connective tissue synthesis and collagen degradation. Sexual as well as nonsexual (e.g. nocturnal penile tumescence) erections may be important in modulating trabecular connective tissue metabolism and to maintain a functional connective tissue/smooth muscle ratio crucial for erectile function[12,13]. This balance of factors that regulate erection may also affect corpus cavernosum structure. Prolonged ischemia or vascular insufficiency may prevent the oxygenation of the corpora that occur during erection, accelerating the processes leading to corporal fibrosis and failure of veno-occlusion.

## EFFECTS OF OXYGEN TENSION OF ERECTION: PATHOLOGICAL IMPLICATIONS OF LOW-FLOW, VENO-OCCLUSIVE PRIAPISM

Priapism is defined as long periods (>6 hours) of prolonged erection[14,15]. High flow, arterial priapism can result from arterial bleeding of the cavernosal artery directly into the corpora cavernosa, resulting in the penis remaining semi-erect to erect all the time[16]. This form of priapism is rare, usually due to trauma and the long-term pathologic consequences are uncertain; primarily due to the few cases reported[16]. In this form of priapism, oxygenation of the corpora cavernosa would be expected to remain high due to the influx of arterial blood bypassing the helicine resistance arterioles. The more common form of priapism is low-flow, veno-occlusive priapism[14,15]. This condition has been associated with sickle cell anemia crisis, penile injection therapy, and/or cocaine use. In this form of priapism, prolonged erection results in increasing hypercapnia, anoxia and acidosis[14,15]. As the blood $PO_2$ falls, ATP production via oxidative phosphorylation is inhibited, decreasing myosin phosphorylation and smooth muscle contractility[14,15,17,18]. Increasing acidosis impairs calcium mobilization, further preventing trabecular smooth muscle re-contraction necessary for detumescence[19]. Both of these factors in combination result in a failure to reachieve smooth muscle tone and prolong smooth muscle relaxation and erection. Timely treatment (<8h) using injections of the alpha adrenergic agonist phenylephrine have been used to restore flaccidity[14]. Recently, digoxin has been proposed as a means of blocking the sodium potassium ATPase and favoring trabecular smooth muscle contractility as a means of managing priapism patients[20]. In those patients in which erection continues 12-24h or more, prolonged anoxia and acidosis can lead to irreversible tissue damage, cell death and corporal fibrosis[14,15,21]. At this point, surgical remediation of the priapism, either through corporal drainage and/or corpus cavernosum/spongiosum shunts or penile implant insertion are necessary[14,15,21]. It is very important to note the distinction in intracorporal condition between erection (pH=7.40, $PO_2$ 90-100mm Hg, trabecular smooth muscle relaxed); flaccidity (pH=7.40, $PO_2$ 25-40mm Hg, trabecular smooth muscle contracted) and low flow, veno-occlusive priapism (pH<7.0, $PO_2$ <5mm Hg, trabecular smooth muscle relaxed, Table 1). The $PO_2$ during flaccidity is obviously sufficient for ATP production necessary to maintain myosin phosphorylation and trabecular smooth muscle contractility[1,17,18].

**TABLE 1**

| State | PO2 | ATP | pH | NO Synthesis | TSM Tone |
|---|---|---|---|---|---|
| Flaccidity | 25 to 40mm Hg | Yes | 7.40 | Greatly reduced | Contracted |
| Erect | 90 to 100mm Hg | Yes | 7.40 | Active | Relaxed |
| V-O-Priapism | Less than 5mm Hg | No | <7.0 | Inactive | Relaxed |
| Arterial H-F-Priapism | ? (75-100mmHg?) | Yes | 7.40 | Active | Relaxed |

Abbreviations: NO, nitric oxide; TSM, trabecular smooth muscle; V-O-Priapism, low flow, veno-occlusive priapism; Arterial H-F-Priapism, high flow, arterial priapism.

## EFFECTS OF OXYGEN TENSION ON VASOACTIVE FACTORS

## NITRIC OXIDE

Nitric oxide is one of the principal vasodilators which regulates penile erection[1,4]. Nitric oxide is a gaseous messenger molecule and is synthesized by the oxidation of a guanidine nitrogen atom of L-arginine to citrulline[1,4,9]. In addition to molecular oxygen and arginine, substrates of the nitric oxide synthase, enzymatic activity requires interaction with calmodulin as well as the cofactor tetrahydrobiopterin. There are three classes of nitric oxide syntheses; type 1 is the constitutive neural NOS typified by the enzyme found in the NANC nerves, type 2 is the constitutive endothelial NOS typified by the enzyme found in vascular endothelium and type 3 is the inducible NOS found in macrophages and smooth muscle[Reviewd in 4]. While the constitutive forms are always present, they are inactive unless intracellular calcium levels increase, bind to calmodulin and activate these enzymes. In this way, the shear stress receptor and the M3 muscarinic acetylcholine receptor on endothelial cells may function by signaling through G-protein coupled pathways (Gi/q) which activate calcium influx or mobilization of calcium stores. Neural nitric oxide synthase has been demonstrated in the pelvic plexus and nerve fibers located in the corpus cavernosum by immunohistochemistry and functional localization with NADPH-diaphorase staining[Reviewd in 4]. Similarly reports of endothelial NOS staining in corpus cavernosum endothelium and smooth muscle has been reported[22]. In these studies, an upregulation of endothelial NOS was seen in aging rabbits. The physiologic significance of endothelial NOS immunoreactivity in trabecular smooth muscle remains to be established.

Nitric oxide potentiates trabecular smooth muscle vasodilation via cGMP dependent and independent pathways[4,23,24]. NO diffuses into the smooth muscle cell, binding to the heme prosthetic group of guanylate cyclase and stimulating cGMP synthesis[4,25]. The increase in cGMP levels eventually leads to sequestration of intracellular calcium, myosin dephosphorylation and smooth muscle relaxation[1,4]. NO has also been shown to act through cGMP independent pathways such as direct activation of the sodium, potassium ATPase[26]. This pathway has been utilized recently in treatment of veno-occlusive priapism patients with digoxin, which inhibits the sodium, potassium ATPase[20].

Since nitric oxide synthesis requires molecular oxygen, it is not surprising that NOS activity is inhibited by low oxygen tension[4,9,25]. Oxygen is a rate-limiting factor for nitric oxide production in the penile corpus cavernosum[4,9]. Penile flaccidity may be maintained in part by inhibition of nitric oxide synthesis. In agreement with this hypothesis, low oxygen tensions has been shown to effect endothelium dependent relaxation of other peripheral vessels in the vasculature[26]. Finally, while nitric oxide elevation of cyclic GMP may not directly affect corpus cavernosum structure[Reviewed in 12], the results of vasodilation of NO or the presence of cytokines, growth factors and vasoactive factors as a result of the actions of NO may play a role in the modulation of corpus cavernosum structure (see below).

## RELEASE OF PROSTANOIDS

Prostanoids are locally acting autacoids which can act both as vasodilators or vasoconstrictors. Prostanoid synthesis requires molecular oxygen; prostaglandin G/H synthase (cyclooxygenase) incorporates 2 molecules of oxygen in its conversion of arachidonate to PGH2[Reviewed in 27]. This precursor is converted into PGE2, PGF2 alpha, prostacyclin (PGI2) and thromboxane A2 (TxA2). Of these prostanoids, PGE2 and PGI2 are potential vasodilators while PGF2 alpha and TxA2 are vasoconstrictors depending on the specific receptors in the target tissue. In the corpus cavernosum, PGE2 relaxes while PGF2 alpha, PGI2 and TxA2 contract smooth muscle strips in organ baths[Reviewed in 1]. It has further been demonstrated that both human and rabbit corpus cavernosum[27] synthesize PGE2, PGF2 alpha, PGI2 and TxA2. Human corpus cavernosum smooth muscle cells in culture produce PGE[5]. A role for PGE has been proposed in erection[5,7,13] whereby as oxygen tension increases and prostaglandin production increases, PGE binds to specific EP2 and/or EP4 receptors on the smooth muscle; elevating cyclic AMP and potentiating smooth muscle relaxation. Decreased oxygen tension inhibits the production of prostanoids in rabbit corpus cavernosum in organ culture as well as PGE production in human corpus cavernosum smooth muscle cells in culture. Despite the decreased synthesis of PGH2 by PGHS during lowered oxygen tension, the available precursor is converted such that there is a decreased production of vasodilating prostanoids and increased production of vasoconstricting prostanoids. Corpus cavernosal ischemia in a rabbit model of atherosclerosis resulted in decreased PGE production and increased thromboxane A2 production (relative to PGE)[Reviewed in 12]. Thus while overall prostanoid synthesis is curtailed, vasoconstricting prostanoids predominate at the lower oxygen tension; further tilting the balance toward corpus cavernosum contractility. This may be particularly important in the production of prostacyclin (PGI2)[28,29]. During erection, there is static pool of blood, due to the low flow, in the corpus cavernosum. It has been suggested that prostacyclin which inhibits platelet aggregation may play an active anticoagulant role to prevent blood stasis during erection[29]. Thus, high oxygen tension favors sufficient prostacyclin and nitric oxide synthesis for proper homeostasis of the corporal blood. During veno-occlusive ischemic priapism the entrapped pool of blood becomes progressively hypoxic; this would inhibit vasodilating prostanoid production, which may account for some of the observed pathological changes, reduction of nitric oxide and PGI2. Although both *in vitro* and *in vivo* studies suggest that ischemia and hypoxemia play a role in the pathology of erectile dysfunction, it is unclear what the ìpoint of no returnî is with ischemic episodes of the corpus cavernosum.

## RELEASE OF ENDOTHELIN

The endothelins are a family of three 21-amino acid peptides which are generated via proteolytic processing of larger precursors. Endothelin 1 isolated from the culture medium of vascular endothelial cells, potently contracts human penile erectile tissue in organ baths[30-33]. All three endothelins bind to trabecular smooth muscle with high affinity (0.4-1nM) and both the isotype specific (ETA) and isotype nonspecific (ETB) receptors are present in corpus cavernosum[31]. Although there are no definitive studies in penile tissue, hypoxic conditions induce the expression of endothelin-1 in human umbilical vein endothelial cells[34]. Endothelin has been proposed as an agent in the pathophysiology of erectile dysfunction; further contributing to smooth muscle contractility in the flaccid state[35-37]. While further studies are necessary to confirm the role of endothelin, it appears that the balance of nitric oxide and endothelin play a role in smooth muscle tone and that lower oxygen tension enhances endothelin expression and inhibits nitric oxide synthase production and gene expression in other vascular beds.

## CHOLINERGIC RECEPTORS

Acetylcholine is neurotransmitter and humoral substance which potentiates trabecular smooth muscle relaxation1. The parasympathetic nervous system suppresses adrenergic tone and may enhance the action of nitric oxide in penile corpus cavernous tissue. Expression of muscarinic acetylcholine receptors have demonstrated in human corpus cavernosum (m1-m4) as well as in cultured human corpus cavernosum smooth muscle cells (m2 and m4)[38]. The m1AChR may be the receptor subtype involved in cholinergic nerves which may also activate NANC neural pathways. Further, there is evidence for m3 ChR on human corpus cavernosum endothelial cells[39]. This receptor which upon stimulation with acetylcholine, may stimulate elevations of calcium and production of nitric oxide via endothelial NOS4. While studies in other vascular tissues demonstrated that lowered oxygen tension alters the effects acetylcholine and may affect receptor levels[40], studies remain to be carried out in human corpus cavernosum.

## ADRENOCEPTORS

Numerous studies have demonstrated the presence of alpha- and beta-adrenergic receptors in penile blood vessels and cavernous smooth muscle[Reviewed in 1,8]. Alpha adrenergic mechanisms are the principal ones ensuring the flaccid state. Three alpha 1 adrenergic receptor subtypes (alpha 1a, alpha 1b and alpha 1d)[41] and all three alpha 2 adrenergic receptor subtypes (alpha 2a, alpha 2b and alpha 2c)[42] are expressed in total human corpus cavernosum biopsies as detected by RNase protection assays. Both alpha 1a and alpha 1d adrenergic receptors as well as a2a and a2c adrenergic receptors are expressed in human corpus cavernosum smooth muscle cells in culture[41-43]. Although the functional significance is unknown, these results suggest that there are postjunctional alpha 2 adrenergic receptors are present on the smooth muscle and further suggest that alpha 1b and alpha 2b adrenergic receptors may be present prejunctionally on adrenergic nerves or on endothelial cells[41-43]. Functional ß-adrenergic receptors are also present in human corpus cavernosum; isoproterenol, a b-adrenergic agonist elevates cAMP in smooth muscle cells[6,7]. Norepinephine binds to all of these classes of adrenergic receptors1 and therefore the response in human corpus cavernosum is a combination of effects from these receptor families. It has been demonstrated that alpha 1 adrenergic tone increases in human corpus cavernosum with age and with vascular disease[44]. While investigations in corpus cavernosum have yet to be performed, prolonged hypoxemia or tissue ischemia selectively increases expression of functionally coupled alpha 1b-adrenergic receptors in arterial but not venous vascular smooth muscle[45]. In contrast, hypoxia followed by reoxygenation has been found to impair alpha 1b-adrenergic receptor function in mesenteric arteries[46]. In chronic oxygen tension, such as in ischemia or hypoxemia, increases local and circulating catecholamines and can induce vascular growth. Surveys of the literature produce what appear to be conflicting effects and point out the need to determine the effects of lowered oxygen tension in a specific vascular bed. Thus, while these findings suggest a role for prolonged low oxygen tension in shifting the balance toward increased adrenergic tone in the corpus cavernosum, definitive studies remain to be done.

## VASCULAR ENDOTHELIAL GROWTH FACTOR (VEGF)

Vascular endothelial growth factor mRNA has been demonstrated to be induced by hypoxia in endothelial cells in culture. A significant increase in VEGF mRNA can be seen as early as three hours after exposure to hypoxia in human umbilical endothelial cells[47]. VEGF increases permeability of the endothelium and can have a number of pleiotopic effects on endothelial cells. While this growth factor has not been investigated in the corpus cavernosum, the effects of hypoxia in other systems suggests a potential role in endothelial injury during prolonged ischemia.

## VASOACTIVE INTESTINAL POLYPEPTIDE

Vasoactive intestinal polypeptide (VIP) is a potent vasodilator in many types of smooth muscle. It stimulates adenylate cyclase and the formation of cyclic AMP[1,48]. There is evidence that VIP may be a neurotransmitter in the corpus cavernosa of different species[49] While a role for VIP in penile erection and detumescence has been proposed[50], no evidence has been presented for effects of hypoxia on VIP or its receptors in the corpus cavernosum.

## CGRP AND SUBSTANCE P

Calcitonin gene-related peptide (CGRP), vasodilator peptide present in pulmonary vascular endothelium[1,51]. CGRP acts in rat pulmonary vessels by an endothelium-dependent mechanism via nitric oxide and hypoxia causes a decrease in the ability of the peptide to dilated pulmonary vessels. While basic mechanism in human penile endothelium are not clear, CGRP may contribute to penile erections in monkeys[52], and a combination of CGRP and PGE1 was used in treatment of erectile dysfunction[53]. Substance P is a potent vasodilators in other vascular tissue, but the action of substance P is not well defined in the human penile tissue[1]. Both CGRP and substance P responses to hypoxia in penile tissue remain to be defined.

## A MOLECULAR PATHOLOGY OF ERECTILE DYSFUNCTION

The penis is comprised only of soft tissue and during erection functions as a blood-filled capacitor of sufficient rigidity at erection for vaginal penetration. The two bodies of erectile tissue, the corpora cavernosa, that are integral to this function are composed of a specialized vascular bed which has a high content of connective tissue (forty eight to fifty five percent)[13,54]. The trabecular smooth muscle cells also synthesize connective tissue which contributes to the structural integrity of the corpora[Reviewed in 55]. A functional trabecular smooth muscle/connective tissue ratio is necessary for veno-occlusion[13,54]. Despite redosing with intracorporeal vasoactive agents, patients with less than approximately thirty seven percent trabecular smooth muscle were more likely to have diffuse leakage[13]. The implications of this finding are that regardless of the amount of trabecular smooth muscle relaxation, veno-occlusion cannot occur in some patients due to higher content of connective tissue and an inability to occlude the draining venules. One area of active research in erectile dysfunction is to identify vasoactive factors, cytokines, autacoids and/or neurotransmitters which may play a role in maintaining the connective tissue/smooth muscle balance[5,12,13,21,54]. In other organ systems, it has been demonstrated that connective tissue can be remodeled under certain conditions. Therefore, it may be possible in some patients for trabecular structure to improve to the point that successful veno-occlusion can occur.

The molecular factors that regulate trabecular smooth muscle structure have recently been investigated[5,12,13,29,54]. Among the potential candidates are transforming growth factor ß1 (TGF-ß1) and prostaglandin E; both of which are synthesized by the trabecular smooth muscle cells[5,13,55]. These vasoactive substances are regulated by oxygen tension; TGF-ß1 is induced under lower oxygen tension conditions consistent with flaccidity while PGE is synthesis is enhanced three fold by higher oxygen tension[5]. In human corpus cavernosum smooth muscle cells in culture, TGF-ß1 can induce a 2.5 to 4 fold increase in collagen synthesis in these cells and this synthesis can be repressed by a single dose of PGE1[55]. In humans[10,11] and in an animal model of erectile dysfunction, there is evidence for a link between prolonged hypoxemia and veno-occlusive dysfunction[56]. In this same animal model, we have demonstrated the relationship between corpus cavernosum structure and function and found that with increasing severity of disease, connective tissue increases with a concomitant decrease in trabecular smooth muscle[57].

While most of the data discussed above are *in vitro* observations, it is interesting to note that nocturnal penile tumescence may provide a daily oxygenation of the corpus cavernosum regardless of sexual activity which may help to maintain a functional trabecular smooth muscle/connective tissue balance[Reviewed in 13]. Normal healthy males have three to five nocturnal erections a night, coincident with rapid eye movement sleep[58]. In conditions where nocturnal penile tumescent episodes are absent (sleep apnea[59]) or disrupted (narcolepsy[60] and epilepsy[61]) erectile dysfunction is more likely to occur. Of particular interest is one study in which thirty three percent of patients with sleep apnea and erectile dysfunction noted improved erectile function as well as nocturnal penile tumescence upon continuous positive airway pressure treatments for their sleep apnea[62]. While research has yet to supply an answer to a number of the questions involved, therapeutic strategies to alter the trabecular structure could be a future means to treat erectile dysfunction.

## EFFECTS OF OXYGEN TENSION ON THE CAVERNOSAL NERVES

The discussion of molecular pathology above centered principally on trabecular smooth muscle. Vascular insufficiency and hypoxemia can result in neuropathy which in turn can have profound effects on erectile function. Smooth muscle relaxation of penile arteries and the corpus cavernosum leading to penile erection, results from sacral parasympathetic neural pathway activation and contraction of smooth muscle and penile arteries controlled by thoracolumbar sympathetic outflow[63]. The sympathetic outflow reaches the penis through two separate anatomical pathways; the prevertebral pathway and paravertebral pathway[63]. Decreased blood flow such as in arterial insufficiencies can lead to ischemic conditions and nerve damage, playing a key role in the etiology of neuropathy. The reduced energy requirement and increased dependence on anaerobic glycolysis appear to be common to neuropathy and hypoxia in other organ systems[64,65]. Unfortunately, there are few studies relating directly to the corpus cavernosum. Therefore, a short review of other organ systems is cited here. In severely hypoxic patients, unmyelinated fiber degeneration in the form of reduced axon density and size with an increase in the number of Schwann cell profiles without axons has also been reported[64]. In patients with chronic respiratory insufficiency, erectile dysfunction of neural origin should be considered and determined[65]. Erectile dysfunction in diabetic patients is traditionally thought to be mainly a consequence of neuropathy[66-68]. Both the parasympathetic and sympathetic innervation may be affected by diabetes[68], because capillary closure and endothelial hyperplasia which occurred in diabetics patients results in hypoxia[69]. Further research is necessary to establish the role of hypoxemia in penile peripheral neuropathy.

## CONCLUSIONS

Erection is a result of hemodynamic event in the penis with a change in oxygen tension in the corpus cavernosum. During the last two decades, significant advances have been made in the understanding of male sexual dysfunction. While much progress has been made, there is very little known regarding the effects of oxygen tension and the mechanisms by which the myriad of humoral substances, vasoactive factors, autacoids, neurotransmitters, cytokines, and growth factors and all of their receptors in the tissue interact to modulate corporal smooth muscle tone and structure. A growing consensus supports the concept that the balance of vasoactive factors regulates trabecular smooth muscle tone and structure and that oxygen tension plays a role in the regulation of these factors. Future research into the mechanisms of physiology and pathophysiology of erectile dysfunction will need to explore not only the effects of oxygen tension on these factors and their receptors but also the crosstalk between these systems which ultimately leads to trabecular smooth muscle tone and the structure of the corpus cavernosum.

## ACKNOWLEDGEMENTS

This work was supported in part by grants DK47950, DK39080 and DK40025 (R.B.M.) from the National Institutes of Health and in part, by a grant from the Mayo Clinic and Foundation.
